# Effect of physical training on airway inflammation in animal models of asthma: a systematic review

**DOI:** 10.1186/1471-2466-13-24

**Published:** 2013-04-24

**Authors:** Vanessa Luks, Andrew Burkett, Lucy Turner, Smita Pakhale

**Affiliations:** 1 Division of Respirology, The Ottawa Hospital, University of Ottawa, Ottawa, Canada; 2 The Ottawa Hospital Research Institute, Ottawa, Canada; 3 Divison of Respirology, The Ottawa Hospital Research Institute, 501 Smyth Road, Ottawa, Ontario K1H 8L6, Canada

**Keywords:** Chronic airway inflammation, Physical exercise, Asthma therapy, Systematic review

## Abstract

**Background:**

There is little data on the effect of exercise on markers of airway inflammation in human asthmatics. The main objective of this review is to determine the effects of physical training on markers of airway inflammation in animal models of asthma.

**Methods:**

A peer reviewed search was applied to Medline, Embase, Web of Science, Cochrane, and DARE databases. Data extraction was performed in a blinded fashion.

**Results:**

From the initial 2336 studies, a total of 10 studies were selected for the final analysis. All were randomized controlled trials with low to moderate intensity training on ovalbumin-sensitized mice. In the exercised group of mice, there was a reduction in BAL eosinophils and Th-2 cytokines, no change in Th-1 cytokines, an increase in IL-10, and a reversal of airway remodeling. The data was not pooled owing to significant heterogeneity between studies, and a funnel plot test for publication bias was not performed because there were few studies reporting on any one outcome measure. The asthma models differed between studies in age and gender of mice, as well as in timing of physical training after sensitization. The risk of bias was unclear for some studies though this may not influence outcome measures. The accuracy of data extracted from graphics is unknown.

**Conclusions:**

Physical training improves airway inflammation in animal asthma models.

## Background

Most clinicians believe that exercise training is beneficial in asthma. Observational studies have shown a correlation between decreased physical activity and the development of asthma [1,2]. Other studies show that exercise training in asthma reduces asthma medications, emergency room visits, symptoms, exacerbations, and can improve lung function, and quality of life [3-9]; however, the 2012 and 2009 Cochrane Collaboration systematic reviews on this topic showed no change in lung function or days without wheeze, although they did show improved cardiopulmonary fitness [10,11]. It is unclear if the benefits of exercise seen in asthmatics result predominantly from a direct impact on airway inflammation, or if they stem from improved cardiac and peripheral muscle conditioning, or both. To expand on this idea, when asked to perform the same preconditioning workload following a period of training, asthmatics have decreased minute ventilation because of improved cardiac and peripheral muscle conditioning. Decreased minute ventilation could reduce exercise-induced bronchoconstriction without ever altering the asthma biology [12]. If exercise does decrease airway inflammation, one would expect a concomitant decrease in non-specific bronchial hyperesponsiveness following exercise, and this is rarely seen [6,13]. Instead, bronchial responsiveness to histamine or methacholine post-training seems to be unchanged in most studies [3,9,14-17].

There is a paucity of studies examining changes in markers of airway inflammation with exercise in human asthmatics; however, a number of such studies have been performed in animal models of asthma. The objective of this review is to determine the effects of physical training on markers of airway inflammation in animal models of asthma.

## Methods

A separate protocol for this review was not previously published.

### Data sources and searches

Studies were identified by searching electronic databases, and scanning reference lists of articles. The search was applied to Medline (1948 – 2012), Embase (1947 – 2012), and adapted for Web of Science (1898 – 2012), Cochrane, and DARE (Database of Abstract of Reviews of Effectiveness). The search strategy was peer reviewed with a librarian at The Ottawa Hospital library. The search was run on April 19^th^, 2012. As we planned to investigate both humans and animal models, the search was not restricted to animal models. No language or region restrictions were applied. The following is the search strategy applied to Medline and Embase: (exp Asthma/ OR asthma.tw) AND (exp Exercise/ or exp Exercise Therapy/ or exp Exercise Movement Techniques/ or exp Physical Conditioning, Animal/ or exp "Physical Education and Training"/ or exp Physical Exertion/ or (exercis$ or aerobic train$ or physical activi$).tw ) AND (inflammation or inflammatory).tw or Inflammation/ or exp Inflammation Mediators/or bronchial biopsy.mp or Cell Count/ or Sputum/ or Adenosine Monophosphate/ or Methacholine Chloride/ or Histamine/ or tumstatin.mp or Basement Membrane/ or Collagen/ or Collagen Type IV/ or Immunoglobulin E/ or T-Lymphocytes, Regulatory/ or eicosanoids/ or leukotrienes/ or prostaglandins/ or thromboxanes/ or cytokines/ or chemokines/ or interleukin-8/ or platelet factor 4/ or interferons/ or interferon type i/ or interferon-gamma/ or interleukins/ or interleukin-1/ or interleukin-2/ or interleukin-3/ or interleukin-4/ or interleukin-5/ or interleukin-6/ or interleukin-7/ or interleukin-9/ or interleukin-11/ or interleukin-12/ or interleukin-13/ or interleukin-15/ or interleukin-16/ or interleukin-17/ or interleukin-18/ or lymphokines/ or leukocyte migration-inhibitory factors/ or macrophage-activating factors/ or transforming growth factor beta/ or tumor necrosis factors/ or tumor necrosis factor-alpha/ or endothelial growth factors/ or fibroblast growth factors/ or platelet-derived growth factor/ or tolloid-like metalloproteinases/ or transforming growth factors/ or cells/ or antigen-presenting cells/ or granulocytes/ or basophils/ or eosinophils/ or neutrophils/ or leukocytes, mononuclear/ or cytokine-induced killer cells/ or killer cells, lymphokine-activated/ or monocytes, activated killer/ or t-lymphocytes, cytotoxic/ or lymphocytes/ or killer cells, natural/ or lymphocyte subsets/ or t-lymphocyte subsets/ or t-lymphocytes/ or cd4-positive t-lymphocytes/ or cd8-positive t-lymphocytes/ or natural killer t-cells/ or monocytes/ or fibroblasts/ or mast cells/ or epithelial cells/ or goblet cells/ or phagocytes/ or histiocytes/ or Nitric Oxide/ or lung inflammation.mp or Th2 Cells/ or Bronchoalveolar Lavage Fluid/ or airway inflammation.mp. or Bronchial Hyperreactivity/).

### Study selection

A scoping search revealed few studies in this area pertaining to either humans or animal models of asthma; therefore, it was decided to investigate this question for both animal models and human asthmatic subjects [18], and to include all trial designs as well as papers published in abstract form only. Subjects had to be animal asthma models. The intervention had to be a physical training program as opposed to a single bout of exercise, and at least one marker of airway inflammation needed to be evaluated at the end of the training program.

The search was applied to each database and the results were combined with removal of duplicates. For the first stage, two authors (V.L. and A.B.) applied inclusion criteria to the titles and abstracts to select out studies. Studies were retained if insufficient information was available to reliably exclude them. For the second stage, the full text of each article was obtained, and both V.L. and A.B. independently reapplied the inclusion and exclusion criteria blinded to the author and publication data. Finally the reference lists of all articles from stage 2 were reviewed by V.L. and A.B. to ensure that all relevant articles had been considered. Disagreements at any stage to include or exclude a study were resolved by consensus with, a third reviewer, S.P.

### Data extraction and quality assessment

After piloting data extraction forms, the data was extracted independently (V.L. and A.B.) blinded to author and publication data. We contacted the study authors to obtain numerical data as the data for the included studies was mostly represented graphically. As numerical data was not available from the authors, graphical data was converted manually into numerical data independently by V.L. and A.B. and compared for consistency. The accuracy of the information was not verified with the authors of the original studies. Authors were successfully contacted to verify if animals used in any of the studies were duplicates to avoid double counting.

Data extraction forms included the following information: study design, characteristics of subjects (type of mouse, age, gender, mass, genetic modifications, specifics of the asthma model), training intervention (modality, intensity, duration of each session, frequency of sessions, duration of training program, timing of training with respect to ovalbumin sensitization), control intervention, markers of airway inflammation that were evaluated, and the change in these markers with exercise.

To determine the validity of the included trials, V.L. applied the Cochrane risk of bias tool to all of the studies included in the review [19].

### Data synthesis and analysis

We grouped studies with similar outcome measures and created forest plots with mean differences, if more than two studies had the same outcome measure.

We converted medians to means where the data appeared normally distributed in the graphs. Using appropriate formulae, we converted interquartile ranges, standard error of the mean, and 95% confidence intervals to standard deviations. Units of measure for outcomes of airway remodeling were different between studies hence a standardized difference in means was applied. After several discussions with experts in the area (see acknowledgements), we decided not to pool the data from the included studies because it was felt that the mice in the different studies were too heterogeneous to be grouped.

Funnel plots were not performed because there were less than 10 studies reporting on any single outcome [20].

## Results

### Search

**Figure 1:**
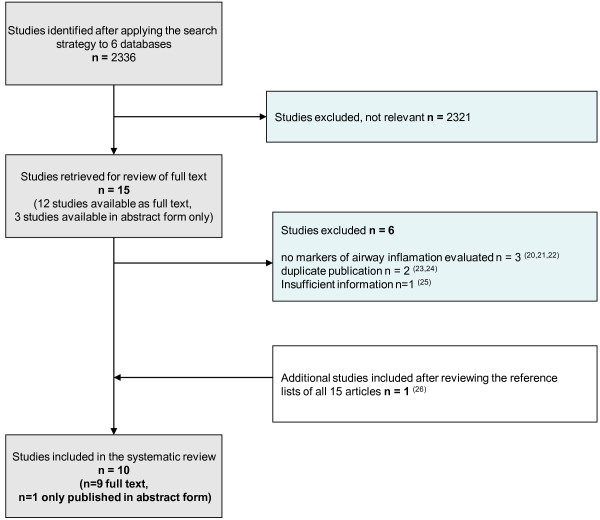
Flow chart of systematic search.

The initial search yielded a total of 2336 citations. Fifteen potentially eligible studies were selected based on title and abstract. We attempted to obtain the full text of these 15 articles; however 3 of them were published in abstract form only. After reviewing the full text of the articles, six studies [21-26] were excluded for reasons stated in Figure 1. One additional study met our inclusion criteria [27] after searching the reference lists of all articles; therefore the total number of studies for the final analysis was 10 (Figure 1).

### Studies

The animals used were BALB/c (Bagg Albino) mice of both genders (see Additional file 1). Essentially two groups of researchers had conducted all studies selected for this review. Mice studied were approximately 3 to 6 weeks of age and of similar mass (18-25 g). The allergic airway models differed, but in general the models were based on sensitization with various doses of intraperitoneal injections of ovalbumin (OVA) adsorbed with aluminum hydroxide, followed by challenge with aerosolized OVA at different frequencies and for an average duration of four weeks. The murine models were allocated to sedentary cage-life or to moderate intensity training 3 to 5 times per week for 4 weeks. One study exercised mice at two intensities of exercise: 50% and 75% of maximum speed reached during a maximal exercise test [28]. As the other studies used 50% VO_2_ max or the equivalent, we chose to include the mice exercised at the lower intensity from that study. Some studies started training after only 1 day of OVA aerosolization [28-31], whereas others waited 7 days [27,32-34].

### Risk of bias

We labeled studies as controlled trials if randomization was not explicitly stated or if we could not confirm randomization by contacting the authors (see Table 1). None of the studies reported on concealment of randomization. Despite these deficiencies, the risk of bias introduced by non-random division of identical mice (gender, age, mass, genetics) is low. One study reported that the researchers who performed the study measurements were blinded to the study group [32]. Hewitt reported that some outcomes were assessed in a blinded fashion [35], and those for which blinding was not described seem to have been performed by an automated machine though this was not confirmed by the authors. Two studies commented on blinding for some measurements but there were other measurements that involved manual counting for which it was unclear if the researcher performing the counting was blinded [33,34]. For all other studies there was no comment regarding blinding. Protocols for individual studies were not available; therefore selective reporting bias remains unknown.

**Table 1:** Risk of bias within studies

**Source**	**Randomization**	**Allocation concealment**	**Blinding of participants & personnel**	**Blinding of outcome assessment**	**Incomplete data outcome**	**Selective reporting**
Dugger et al. 2010	Low risk	Low risk	Low risk	Unclear risk	Unclear risk	Unclear risk
Hewitt et al., 2010	Low risk	Low risk	Low risk	Low risk	Unclear risk	Unclear risk
Lowder et al., 2010	Low risk	Low risk	Low risk	Unclear risk	Unclear risk	Unclear risk
Pastva et al., 2005	Low risk	Low risk	Low risk	Unclear risk	Unclear risk	Unclear risk
Pastva et al., 2004	Low risk	Low risk	Low risk	Unclear risk	Unclear risk	Unclear risk
Silva et al., 2010	Low risk	Low risk	Low risk	Low risk	Unclear risk	Unclear risk
Vieira et al., 2011	Low risk	Low risk	Low risk	Low risk	Low risk	Unclear risk
Vieira et al., 2009	Low risk	Low risk	Low risk	Low risk	Low risk	Unclear risk
Vieira et al., 2008	Low risk	Low risk	Low risk	Low risk	Low risk	Unclear risk
Vieira et al., 2007	Low risk	Low risk	Low risk	Low risk	Low risk	Unclear risk

### Results of individual studies

**Figure 2:**
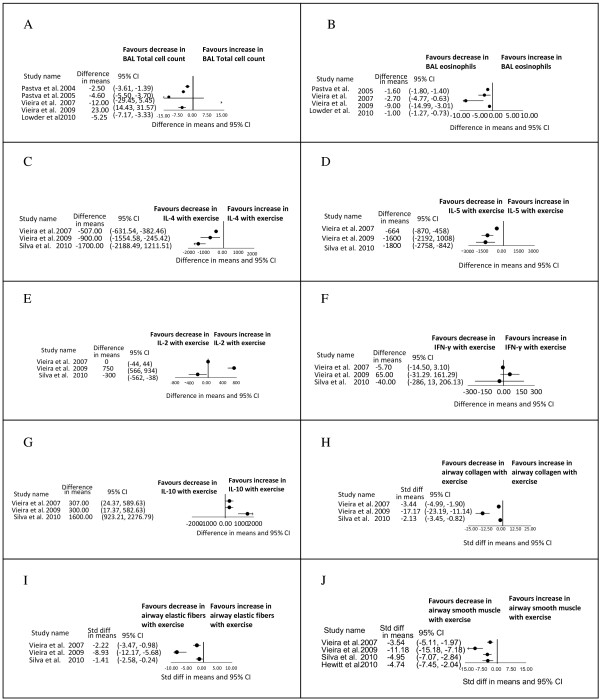
**Forest plots of results. A)** Bronchoalveolar lavage total cell counts (cells* 10^5^). **B)** Bronchoalveolar lavage eosinophils (cells* 10^5^). **C)** Peribronchial density of cells positive to Interleukin-4 (Il-4) (cells/mm^2^). **D)** Peribronchial density of cells positive to Interleukin–5 (Il-5) (cells/mm^2^). **E)** Peribronchial density of cells positive to Interleukin-2 (Il-2) (cells/mm^2^). Note: in the graphical data presented in the original paper by Silva et al. 2010, there is no change in IL-2 with exercise, but our conversion to numerical data was imperfect and makes it look as if there is a change when in fact there was not. **F)** Peribronchial density of cells positive to Interferon-γ (IFN- γ) (cells/mm^2^). Note: in the graphical data presented in the original paper by Vieira et al. 2009, there is a significant increase in IFN-γ, but our conversion to numerical data was imperfect and makes it look as if there is no significant change, when in fact there was a significant change. **G)** Peribronchial density of cells positive to Interleukin-10 (Il-10) (cells/mm^2^). **H)** Airway collagen fibers (Silva: % area, Vieira 2007: % volume, Vieira 2009: % volume). **I)** Airway elastic fibers (Silva: % area, Vieira 2007: % volume, Vieira 2009: % volume). **J)** Airway smooth muscle fibers (Silva: %, Vieira 2009: index, Vieira 2007: area, Hewitt: microns).

The forest plot (Figure 2A) demonstrates that there was a decrease in the total cell count in the trained group in 3 out of 5 studies [27,33,34]; the total cell count was unchanged in one study [28], and increased in another [30]. The study in which the cell count was increased with exercise was attributable to increases in the BAL neutrophils, macrophages, and lymphocytes with exercise. This was not explained by the authors and was not seen in similar experiments by the same authors on different mice.

BAL eosinophils in the trained group decreased in all studies [27,28,30,33,34]. The data from Pastva 2004 is not included in the forest plot (Figure 2B) because the data could not be converted to a numerical format; however, the figure legend in the original study does indicate a significant decrease in the BAL eosinophils in the trained compared to the sedentary group of OVA-sensitized animals.

Vieira et al. demonstrated that training decreased eosinophils in the lung parenchyma and pulmonary perivascular tissue [31]. Three studies showed a decrease in peribronchial density of eosinophils with training [28,30,32].

Vieira et al. and Pastva et al. reported decreases in vascular cell adhesion molecule 1 (VCAM-1) with training, but results were discordant for intercellular adhesion molecule 1 (ICAM-1) [27,29,33]. CXC chemokine KC (the murine analogue of interleukin-8 and involved in chemotaxis of neutrophils and primed eosinophils), was decreased in BAL fluid following training [27,33]. Chemokine ligand 5 (CCL 5), also known as RANTES (Regulated upon Activation Normal T cell Expressed and Secreted), as well as CCL 11 (also known as eotaxin-1), both involved in eosinophil recruitment, were decreased in the trained group [29].

Airway immunohistochemistry showed uniform decreases in expression of the Th2 cytokines (IL-4, IL-5, IL-13) in the trained group of mice in all studies reporting these outcomes [28,30,32] (Figure 2C-D). Pastva et al. also reported a decrease in IL-4, and IL-5 in BAL [33]. Two out of three studies reporting on Th1 cytokine expression in airways showed no change in IL-2 or IFN-γ with training suggesting that the benefits of training are not a result of a shift to a Th1 immune response [28,32] (Figure 2E-F). The study that did show an increase in the Th1 cytokines with training involved mice exposed not only to OVA, but also to creatine [30]. It is important to note in Figure 2 E and F that when manually converting the graphical data presented in the original papers by Silva 2010 and Vieira 2009 to numerical data, for the purpose of creating forest plots, there is inherent error. As a result, the forest plot for IL-2 for Silva 2010 (Figure 2E) gives the impression of a significant decrease in IL-2 but in fact there was no change in the original paper. Similarly, the forest plot for IFN-γ for Vieira 2009 gives the impression of no significant increase in IFN-γ, when in fact the increase was significant in the original paper. All three of these studies however demonstrated that IL-10, an anti-inflammatory cytokine [36], increased in the trained group [28,30,32] (Figure 2G). Silva et al. also showed that, interleukin -1 receptor antagonist, an anti-inflammatory cytokine, increased after training [32,37,38].

Vieira et al. reported the effect of training on perivascular expression of Th2 and Th1 cytokines to be identical to the changes at the level of the airway (decreased IL-4, IL-5; increased IL-10, no change in IL-2 or IFN-γ) [31]. Expression of these cytokines post-training was the same in the lung parenchyma with the exception of an increase in IL-2 [31]. In addition, there was decreased expression of the monocyte chemotactic protein -1 (MCP-1), a potent stimulator of histamine and leukotriene release in human asthmatic airways [39], in the perivascular and parenchymal compartments.

Of the three studies reporting immunoglobulins, all showed no effect of training on total IgE or IgG1 [28,32,33]. Only one of these studies showed decreased OVA-specific IgE [33].

Four studies reported on nuclear factor-κB (NF-κB). Vieira et al. and Silva et al. demonstrated decreased peribronchial expression of NF-κB in the trained asthmatic mice [29,32]. Vieira et al. demonstrated decreased perivascular and parenchymal NF-κB p65 [31]. Pastva et al. demonstrated decreased NF-κB subunit p65 translocation in the trained mice and decreased IκB alpha phosphorylation (decreased activation of NF-κB) [33], as well as decreased DNA binding activity [27].

Four studies [28,30,32,35] reported on airway remodeling and showed that training reduced collagen fibers, elastic fibers, and smooth muscle content (Figure 2H-J). Two studies also reported decreased epithelial hypertrophy with training [28,33]. Pastva et al. and Vieira et al. also showed decreased airway goblet cells with training [27,29], but there were inconsistent results of the effect of training on mucin production [27,29,32]. Vieira et al. alone reported on the effect of training on vascular and parenchymal remodeling and showed decreased perivascular edema, decreased perivascular collagen, elastic fibers, and smooth muscle thickness mirroring the airway changes [31]. No changes occurred after training in the parenchymal compartment of the murine lungs [31].

Vieira et al. showed reduced expression of epithelial insulin-like growth factor 1 (IGF-1), epidermal growth factor receptor (EGFr), vascular endothelial growth factor (VEGF), and transforming growth factor – beta (TGF-β) post-training [29]. These researchers also demonstrated decreased IGF-1 in perivascular and parenchymal tissue post training [31]. Vieira et al. also showed decreased matrix metalloprotease -12 (MMP-12) and tissue inhibitor of metalloprotease 2 (TIMP-2) in airway epithelium of trained animals.

Lowder et al. showed increased CD4+CD25+ Treg cells in the lungs and mediastinal lymph nodes draining the lungs in exercised asthmatic animals as opposed to controls. They also showed that such Treg cells isolated from the lungs, mediastinal lymph nodes, and spleens of exercised as opposed to sedentary asthmatic mice, had superior in vitro suppression of Th1 and Th2 cytokine responses as well as CD4+CD25- proliferation [34]. In contrast, Silva et al. showed that Foxp3, a transcription factor expressed specifically in CD4+CD25+ Treg cells, was unchanged by OVA-sensitization or training [32]; however the method of detection was very different from that of Lowder et al.

A single study showed decreased expression of purinergic receptor 7 (P2X7R) in trained animals. Vieira et al. reported decreased markers of oxidative and nitrosative airway stress (GP91 phox, 3-nitrotyrosine, and 8-isoprostane) in the exercised group of asthmatic mice compared to the sedentary group but no change in antioxidant enzymes (superoxide dismutase 1 and 2, and glutathione peroxidase) [29].

Hewitt et al. demonstrated decreased airway resistance after methacholine challenge and increased circulating epinephrine in trained as opposed to sedentary murine asthma models [35]. Although there was no change in β2 agonist receptor expression on airway smooth muscle, there was decreased prostaglandin E2 (PGE2) in BAL fluid and decreased G protein receptor kinase 2 (GRK) expression in the smooth muscle cells.

## Discussion

In summary, we conducted a systematic review, and identified and reviewed ten studies examining the effect of exercise training in murine models of asthma. The findings include a decrease in BAL eosinophilia, a decrease in Th2 cytokines, an increase in IL – 10, and reversal of airway remodelling in trained compared to sedentary murine asthma models. Other interesting findings included decreased expression of NF-κB, a family of transcription factors promoting the expression of genes for cytokines [40], a decrease in growth factors, which are hypothesized to be involved in the abnormal repair process [39,41,42], a decrease in P2X7R, a receptor involved in the control of pro-inflammatory cytokines that is upregulated in OVA-sensitized mice [29], decreased reactive nitrogen and oxygen species, decrease in desensitization of β2 agonist receptors (via decreased PGE2 and GRK), and an increase in pulmonary Treg cells with superior suppression of lymphocyte proliferation. Previous studies have shown that CD4+CD25+ Foxp3+ Treg cells inhibit the development and progression of allergic diseases including asthma [43].

The limitations of this review include those related to accuracy of results, assessment of bias within studies, heterogeneity between studies, and applicability to human subjects. More importantly, we do not know if there was any publication bias.

The accuracy of data extracted from the studies was compromised as we were unable to obtain numerical data from the authors. Furthermore, differential cell counts were performed manually which is problematic because of the effects of different sample handling and inter-observer variability. We were also unsuccessful in our attempts to ascertain for certain studies whether outcome assessors were blinded, and which outcomes were automated by machines making the risk of bias within studies unclear.

The studies were quite heterogeneous in the types of mice used, the allergic airway models employed, and the training regimes. In addition, there may have been many other differences between the mice in each study such as where they were bred (about half of the studies obtained mice from The Jackson laboratory, but other studies did not state the facility of origin), and how they were handled. Mice from one facility can react differently from those bred in another facility, and mice are very sensitive to how they are handled. These may explain some of the inconsistencies in outcomes between studies.

The murine models of asthma employed in these studies and others do not perfectly reflect the airway changes or immunologic responses in human asthma. Firstly, murine models have parenchymal and vascular inflammation not seen in human asthmatics. Other important differences include the fact that murine eosinophils are less likely to degranulate, murine mast cells release mediators not seen in human mast cells, the Th1 versus Th2 responses are much more polarized in mice than in humans, and the baseline anatomical features of the airways differ greatly between mice and humans [44,45].

Asthma of course does not exist naturally in mice so these models are more appropriately referred to as models of chronic allergic inflammation. Many methods of how best to achieve such a model have been published as studies and in reviews [45-50]. The sensitization process, antigen employed, frequency and duration of aerosolization, and specific species are all important in determining the type and timing of the allergic response. If not all the features of chronic airway inflammation are present at the time of initiation of aerobic training, this could affect the results [46,47,50]. Similarly, it has been observed in some models that extended exposure to aerosolizations can lead to tolerance and reduced airway inflammation over time [46,49,51]; however, chronic changes including airway hyperresponsiveness, smooth muscle hypertrophy, mucous hypersecretion and fibrosis were seen to persist for eight weeks after the last aerosolization in other protocols describe by Kariyawasam [47]. Clearly the method of inducing “asthma” in animals and the timing of intervention can affect the outcomes.

After our final searches for this review (April 2012) there was a study by Olivo et al. [52] published in July 2012 studying the effects of aerobic exercise on guinea pig models of asthma, considered by some to be a closer model to human asthma. This study demonstrated that in the OVA sensitized guinea pigs with 8 weeks of aerobic exercise there was a reduction in the following compared to the sedentary sensitized group: peribronchial density of eosinophils and lymphocytes, peribronchial expression of interleukin IL-4 and IL-13, and airway edema. Similar to the murine models in this review, exercise did not affect the Th1cytokines (Il-2 and IFN-γ), IgE, or IgG1. Unlike the murine models reviewed in this paper, exercise did not increase IL-10 or attenuate the increase in smooth muscle. Furthermore, exercise seemed to increase epithelial cell thickness and had no effect on exhaled nitric oxide. As alluded to in the previous paragraphs, animal models attempt to mirror the pathologic changes seen in humans. It is important to note that the mechanism of action of achieving features of allergic airway inflammation may be different in different species, and similarly, the mechanism by which exercise attenuates the inflammation may differ between species. This might explain why levels of IgE in murine and guinea pig asthma models correlate poorly with airway changes and do not seem to decrease with the other beneficial changes of training [51], and perhaps why there are differential effects of exercise on IL-10 and smooth muscle between sensitized guinea pigs and mice, aside from other technical explanations hypothesized by the authors.

The most important limitation of this review is that markers of airway inflammation are surrogate outcomes rather than practice-changing outcomes of relevance to clinicians; however, the results of this review do add to our understanding of the effects of exercise on asthmatic airways. The results of this review suggest that exercise not only improves cardiovascular fitness, but also directly reduces airway inflammation in animal asthma models.

## Conclusions

Physical training in murine models of asthma results in decreased expression of several markers of airway inflammation. Despite limitations, there is evidence to support similar studies to verify this effect in human asthmatics.

## Competing interests

Dr. S. Pakhale is supported by the Ottawa Hospital Research Institute and the Department of Medicine, The Ottawa Hospital, Ottawa, Canada. There was no role of the funders in conducting this systematic review. The authors declare that they have no competing interests.

## Authors’ contributions

SP had full access to the data and takes full responsibility for the integrity of the data and the accuracy of the data analysis. SP: contributed to the concept, design, implementation, statistical analysis, interpretation and writing; VL: contributed to the data management, statistical analysis, interpretation and writing; AB: contributed to the data management, statistical analysis and writing; LT: contributed to the data analysis, interpretation and writing. All authors read and approved the final manuscript.

## Pre-publication history

The pre-publication history for this paper can be accessed here:

http://www.biomedcentral.com/1471-2466/13/24/prepub

## Supplementary Material

Additional file 1 **Summary of included studies.** This file provides details of the studies included in this systematic review including the number of subjects, animal species, asthma model, study design, intervention, and outcome measures. Click here for file
