# Supplementary material for: Effect of physical training on airway inflammation in animal models of asthma: a systematic review
Source: BMC Pulm Med. 2013 Apr 24;13:24. doi: 10.1186/1471-2466-13-24 (PMC3691924; doi:10.1186/1471-2466-13-24)
Supplement: Additional file 1 — Summary of included studies. This file provides details of the studies included in this systematic review including the number of subjects, animal species, asthma model, study design, intervention, and outcome measures. [file 1471-2466-13-24-S1.pdf]

Summary of included studies

Source	Number of subjects	Animal	Asthma model	Study design	Intervention	Outcomes
Dugger et al. 2010	unknown	BALB/c with CD4+ Thy1.2+ cell from the D011.10-T^{LUX} model<br>sex unknown<br>age unknown	“OVA sensitization/challenge”	CT	Treadmill at 13.5 m/min , 0% grade (no other information stated)	-T helper cell CCR7, CCR8, CXCR3, and CCR4 expression, function, and migration in/out of the lung
Hewitt et al., 2010	8-16	BALB/cJ<br>females<br>age 3 – 5 weeks	OVA-alum IP days 0, 14; then aerosolized OVA (concentration unclear) days 21 – 25 & day 28	CT	Treadmill 45 – 60 min 3x/week for 4 weeks at 13.5 m/min, 0% grade; in addition, 15 min total warm-up/cool-down per session. Started 1h post OVA treatment on day 28	-serum epinephrine<br>-BAL: PGE_2<br>-lung histology: airway smooth muscle thickness<br>-ex-vivo airway smooth muscle cells analyzed for β_2 AR , GRK-2, EP1<br>-airway resistance to increasing amounts of methacholine
Lowder et al., 2010	6-12	C.Cg-$Foxp3^{tm2Tch}$/J reporter on BALB/cJ background<br>females<br>age 5 – 6 weeks	OVA-alum 50 mcg days 0, 14; then aerosolized OVA at 5 mg/mL 30 min/day x 5 days for day 21 – 25, then 10 min 3 days/week for the remainder	RCT	Treadmill for 45 min 3x/wk for 4 wks at 10 – 13.5 m/min, 0% grade. Started 1h post-OVA on day 28	-BAL: total cells, differential<br>-Lung flow cytometry for % Foxp3+ cells<br>-in vitro suppression of effector cell proliferation by CD4+CD25+Foxp3+ cells from the lungs, mediastinal lymph nodes, spleen<br>-in vitro levels of lung/spleen Il-4, Il-5, TGF-β, Il-10, Il-17 in the supernatents of spleen cultures at an effector:suppressor ratio of 1:1
Pastva et al., 2005	10-12	BALB/cJ<br>females<br>age 3 – 5 weeks	OVA 50 mcg IP days 0, 14; then aerosolized OVA at 5 mg/mL for 30 min/day x 5 days starting day 21, thereafter boosters of aerosolized OVA at the same concentration 10 min/day 5days/wk	RCT	Treadmill: 1st week 10m/min x 30 min 3x/wk, thereafter 45 min at 13.5 m/min 0% grade 3x/week for 3 weeks; in addition 15 min total warm-	-BAL: total cells, eosinophils, CXC chemokine KC, VCAM-1<br>-lung immunofluorescence: NF-κB DNA binding activity, NF-κB nuclear translocation

					up/cool-down. Started 1-2h post OVA on day 28.	
Pastva et al., 2004	10-18	BALB/cJ females age 3 – 5 weeks	OVA- alum 50 mcg IP days 0, 14; then aerosolized OVA 5mg/mL starting day 21 for 30min/day x 5days, then 10min/day 5days/wk	RCT	Treadmill for 45 min 3x/wk for 4 weeks at 10 m/min 0% grade x 2 weeks, then 13.5m/min 0% grade x 2 weeks; in addition, 15 min total of warm-up/cool down per session. Started 2h post OVA on day 28.	-serum: total IgE , OVA-specific IgE<br>-BAL: total cells, differential, total protein, KC, MCP-1, RANTES, Il-4, Il-5<br>-lung histology : peribronchial lymphocytes, perivascular lymphocytes, epithelial hypertrophy, goblet cells/mucin, index of inflammation, immunohistochemistry for VCAM-1, NF-κB p65
Silva et al., 2010	14	BALB/c males age 4 weeks	OVA-alum 10 mcg IP days 0, 14, 28, 42; then 1% aerosolized OVA starting day 21 for 30 min 3x/week until day 54	CT	Treadmill for 60 min 5x/wk for 4 weeks at 50% max speed based on a maximal exercise capacity test. Started day 28	-serum IgE and IgG_1<br>-peribronchial histology: eosinophils , mucous production, airway smooth muscle thickness, airway collagen deposition, airway elastic fiber deposition<br>-peribronchial immunohistochemistry:, CD3+ T cells, CD4+ T cells, Il-4, Il-5, Il-13, Il-2, IFN-γ, Fox p3, Il-10, Il-1ra, NF-κB
Vieira et al., 2011	16	BALB/c males age unclear	OVA-alum 20 mcg IP days 0, 14, 28, 42; then 1% aerosolized OVA starting at day 21 3x/week until day 50	CT	Treadmill for 60 min 5 x/week for 4 weeks at 50% max speed based on a maximal exercise capacity test. Started 1 day post first OVA aerosolization.	-BAL: total cells, eosinophils, epithelial cells<br>-airway tissue histology: goblet cells, neutral mucin , ciliated cells<br>-airway immunohistochemistry: Il-13, Il-14, Il-5, VCAM-1, CCL11, CCL5, ICAM-1, eNOS, nNOS, iNOS, Il-2, IFN-γ, NF-κB , Il-10, SOD1, SOD2, GPX,

						GP91phox, 3-Nitrotyrosine, 8-Isoprostane, IGF-1, EGFr, VEGF, TGF-β, MMP-12, TIMP-1, TIMP-2, MMP-9, P2X7R
Vieira et al., 2009	16	BALB/c<br>males<br>age unclear	OVA-alum 10 mcg IP days 0, 14, 28, 42; then 1% aerosolized OVA starting day 21 for 30 min 3x/wk up to day 53 Both groups also given oral creatine supplementation (0.5g/kg 5x/week from day 21 to 53).	CT	Treadmill for 60 min 5x/week for 4 wks at 50% max speed based on a maximal exercise capacity test. Started 1 day post first OVA aerosolization.	-BAL: total cell count, differential<br>-airway histology: peribronchial density of eosinophils, airway volume proportion of collagen & elastic fibres, airway smooth muscle index, bronchoconstriction index<br>-airway immunohistochemistry: Il-4, Il-5,Il-2, IFN-γ, Il-10
Vieira et al., 2008	16	BALB/c<br>males<br>age unclear	OVA –alum 20 mcg IP days 0, 14, 28, 42; then 1% aerosolized OVA 3x/week days 21 - 50	CT	Treadmill for 60 min 5 x/week for 4 weeks at 75% max speed based on baseline maximal exercise test. Started 1 day post first OVA aerosolization.	-lung histology: perivascular eosinophils & mononuclear cells, parenchymal eosinophils & mononuclear cells, volume proportion of elastic & collagen fibres in the artery wall and in lung parenchyma<br>-lung immunochemistry: perivascular expression of Il-4, Il-5, Il-2, IFN-γ, MCP-1, IGF-1, Il-10, NF-κB p65, parenchymal expression of Il-4, Il-5, Il-2, IFN-γ, MCP-1, IGF-1, Il-10, NF-κB
Vieira et al., 2007	16	BALB/c<br>males<br>age unclear	OVA-alum 20 mcg IP days 0, 14, 28, 42; then 1% aerosolized OVA 3x/week days 21 - 50	CT	Treadmill for 60 min 5 x/week for 4 weeks at 50% max speed based on baseline maximal exercise test. Started 1 day post first OVA aerosolization.	-serum IgE, IgG1 titres<br>-BAL : total cell count, eosinophils, PMNs, mononuclear cells<br>-airway histology: peribronchial density of mononuclear cells, eosinophils , volume proportion of collagen,

						elastic fibres , smooth muscle area, epithelial thickness, bronchoconstriction index<br>-airway immunohistochemistry: Il-4, Il-5,Il-2, Il-10, IFN-γ

BALB : Bagg Albino (inbred research mouse strain), OVA : ovalbumin, CT : controlled trial, CCR : chemokine (C-C motif) receptor, CXCR : CXC chemokine receptor, alum : aluminum hydroxide, IP : intraperitoneal, BAL : bronchoalveolar lavage, PGE_2 : prostaglandin E2, β2AR : beta-2 adrenergic receptor, GRK2 : G-protein receptor kinase 2, EP1 : E prostanoid-1 receptor, Treg : regulatory T cell–specific transcription factor, RCT : randomized controlled trial, CD : cluster of differentiation, Il : interleurkin, TGFβ : transforming growth factor-beta, KC : keratinocyte chemoattractant, VCAM-1 : vascular cell adhesion molecule-1, NF-κB p65 : a subunit of the nuclear factor-kappa B transcription complex, IgE : immunoglobulin E, MCP-1 : monocyte chemotactic protein-1, RANTES : regulated upon activation normal T cell expressed and secreted, IgG1 : immunoglobulin G1, CCL : chemokine (C-C motif) ligand, ICAM-1 : intercellular adhesion molecule-1, eNOS : endothelial nitric oxide synthase, nNOS : neuronal nitric oxide synthase, iNOS : inducible nitric oxide synthase, IFN-γ : interferon-gamma , SOD1 : superoxide dismutase-1, GPX : glutathione
